# Infectivity and Progression of COVID-19 Based on Selected Host Candidate Gene Variants

**DOI:** 10.3389/fgene.2020.00861

**Published:** 2020-09-04

**Authors:** Gayatri R. Iyer, Sayani Samajder, Syeda Zubeda, Devi Soorya Narayana S, Vishakha Mali, Sharath Krishnan PV, Anuradha Sharma, Neyha Zainab Abbas, Nandini Shyamali Bora, Amulya Narravula, Qurratulain Hasan

**Affiliations:** ^1^Department of Genetics and Molecular Medicine, Kamineni Hospitals, Hyderabad, India; ^2^Department of Genetics, Osmania University, Hyderabad, India; ^3^Department of Genetics, Kamineni Hospitals, Hyderabad, India

**Keywords:** COVID-19, candidate genes, variants, polymorphisms, infectivity

## Abstract

**Introduction:** Severe Acute Respiratory Syndrome Coronavirus-2 (SARS-CoV-2) has spread around the globe. Susceptibility has been associated with age, biological sex, and other prior existing health conditions. However, host genes are involved in viral infectivity and pathogenicity, and polymorphisms in these could be responsible for the interethnic/interindividual variability observed in infection and progression of COVID-19.

**Materials and Methods:** Clinical exome data of 103 individuals was analyzed to identify sequence variants in five selected candidate genes: *ACE2, TMPRSS2, CD209, IFITM3*, and *MUC5B* to assess their prevalence and role to understand the COVID-19 infectivity and progression in our population.

**Results:** A total of 497 polymorphisms were identified in the five selected genes in the exomes analyzed. Thirty-eight polymorphisms identified in our cohort have been reported earlier in literature and have functional significance or association with health conditions. These variants were classified into three groups: protective, susceptible, and responsible for comorbidities.

**Discussion and Conclusion:** The two polymorphisms described in literature as risk inducing are rs35705950 in *MUC5B* gene and *TMPRSS2* haplotype (rs463727, rs34624090, rs55964536, rs734056, rs4290734, rs34783969, rs11702475, rs35899679, and rs35041537) were absent in our cohort explaining the slower infectivity of the disease in this part of India. The 38 functional variants identified can be used as a predisposition panel for the COVID-19 infectivity and progression and stratify individuals as “high or low risk,” which would help in planning appropriate surveillance and management protocols. A larger study from different regions of India is warranted to validate these results.

## Introduction

Severe Acute Respiratory Syndrome Corona virus-2 (SARS-CoV-2) is a new virus responsible for an outbreak of respiratory illness, since December 2019, named as COVID-19, which has spread to several countries around the globe. Susceptibility initially was associated with age, biological sex, and other prior existing health conditions, which is true for all infectious diseases (Yi et al., 2020). The World Health Organization has declared it as a Public Health Emergency of International Concern. Although it is still early to predict susceptible populations, this needs to be addressed urgently to triage and safeguard individuals with high risk of infectivity and/or the potential to get this progressive disease with adverse prognosis.

SARS-CoV-2 belongs to the family of RNA viruses known as coronaviruses. Sequencing results from patient isolates have indicated that SARS-CoV-2 is similar to the beta (β) coronaviruses identified in bats. Two subtypes of coronaviruses have earlier been responsible for large-scale pandemics, Severe Acute Respiratory Syndrome (SARS) and Middle East Respiratory Syndrome (MERS). However, COVID-19 has been found to have higher levels of transmissibility than the earlier two viruses (Adhikari et al., 2020).

It has been observed that susceptibility to COVID-19 shows geographical variation, and it has also been noted that individuals have different disease severities, indicating that host genomic variations might be playing an important role. These need to be determined to predict disease risk and outcome, as well as help in plan new specific interventions and vaccine delivery.

A single patient follow-up study from Australia indicated that robust multi-factorial immune responses can be elicited to SARS-CoV-2, which is similar to the avian H7N9 disease, suggesting that early adaptive immune responses might correlate with better clinical outcomes (Thevarajan et al., 2020).

Recent studies have found that the SARS-CoV-2 and SARS-CoV genomes share around 80% of homology and use the same cell entry receptor, angiotensin converting enzyme 2 (*ACE2*), for infectivity (Gralinski and Menachery, 2020). Although there is no direct evidence that supports that variants of *ACE2* exhibit differential binding of SARS-CoV-2 with S-protein, 11 common variants and one rare variant (rs143695310) associated with high expression of *ACE2* in tissues was reported by (Cao et al., 2020). There is only one report from the Indian population about polymorphisms and their *in-silico* functional significance (Sharma et al., 2020).

After analyzing data from > 200,000 exomes, homozygous/heterozygous/hemizygous loss of function mutations of *ACE2* appear to be extremely deleterious (Cirulli et al., 2020). Although rare, some of these variants affect the regions that interact wit h SARS-CoV-2, and further research may identify some of these variants as conferring resistance or heightened susceptibility to the virus.

Binding of SARS-CoV S protein to *ACE2* triggers subtle conformational rearrangements, which are believed to increase the sensitivity of the S protein to proteolytic digestion at the S1 and S2 subunits (Haga et al., 2008). Cleavage of the S protein by host cell proteases is essential for viral infectivity and the responsible enzyme is the type II transmembrane serine proteases (*TMPRSS2*) (Matsuyama et al., 2010).

*TMPRSS2* is expressed in *ACE2*-positive cells, including the human lung, and results obtained with surrogate cell culture systems suggest that *TMPRSS2* might play a significant role in coronavirus spread in the human respiratory tract (Bertram et al., 2013). *TMPRSS2* facilitates infection via two independent mechanisms, cleavage of *ACE2*, which might promote viral uptake, and cleavage of SARS-S, which activates the S protein for membrane fusion (Heurich et al., 2014).

Viruses use multiple alternative receptors to enter host cells and a study by Cai (2020) reported that apart from *ACE2*, dendritic cell-specific intercellular adhesion molecule-3-grabbing non-integrin (DC-SIGN) known as *CD209* may be important for microbial infectivity (Cai, 2020). DC-SIGN acts as an adhesion molecule and also initiates innate immunity, although the exact mechanism is not clear, but it is known to be involved in microbial clearance through capture, destruction, and presentation of antigens. High expression of DC-SIGN in older individuals and higher gene expression of L-SIGN in Caucasians when compared to Asians was reported. It was also shown that significantly higher DC-SIGN gene expression occurs in the lungs of smokers, especially former smokers, indicating that this may affect SARS-CoV-2 infection (Cai, 2020). It is commonly believed that RNA virus entry into cells by endocytosis is regulated with interferon (IFN)-induced transmembrane (IFITM) proteins, which detect and eliminate viral invaders before the establishment of infection (Weidner et al., 2010). Therefore, mutation in the genes coding for these proteins can lead to variability in establishing infections in the host. *IFITM3* is a cellular restriction factor that inhibits infection of influenza virus and many other pathogenic viruses, and sequence variants of this gene exhibits ethnic differences (Yount et al., 2010; Zhang et al., 2013) This highlights the significant role of *IFITM3* genetic variants on the epidemiology of influenza. The *IFITM3* rs12252 polymorphism has different allele frequencies among different ethnicities ranging from 0% among Japanese to 44% among Chinese populations. Zhang et al. (2013) found a significant association between rs12252 and susceptibility to severe, but not mild flu, among Asians and evidence of an association between rs12252 C allele homozygotes and susceptibility to mild flu in patients with Caucasian ancestry (Zhang et al., 2013).

Respiratory surfaces are exposed to pathogens continuously and a protective mucus barrier traps and eliminates them via mucociliary clearance. *MUC5B* is an evolutionarily conserved gene that encodes the principal macromolecules in airway mucus (Roy et al., 2014). Genetic variants of *MUC* genes are linked to diverse lung diseases. *MUC5B* deficiency causes organisms to accumulate in upper and lower airways and is responsible for development of idiopathic pulmonary fibrosis (Kaur et al., 2017). *MUCB5* variants may also be linked to COVID-19 progression. A gain-of-function promoter polymorphism, rs35705950 of *MUC5B* has been indicated to be responsible for increased severity of lung disease. This variant has been considered to confer the largest risk, genetically for pulmonary fibrosis (Roy et al., 2014; Helling et al., 2017).

The aim of the present study was to identify variants in these five selected candidate genes from the clinical exome data available with us for more than 100 individuals and make an attempt to classify them as relevant to the present COVID-19 aetiopathology, especially for the Indian population.

## Methods

We performed a literature survey to study the global trends of COVID-19 infection and its severity in different populations to understand and predict the impact of the pandemic in the Indian population. Scientific reports, publications, and Genome Wide Association Studies (GWAS) results were studied with respect to severe acute respiratory conditions both viral and non-viral. In view of limited data on COVID-19 susceptibility studies, we included studies on other pandemics like H1N1, H7N9, and MERS, and we identified five candidate genes and their polymorphisms, which may be responsible for infectivity, disease progression, and disease outcome. The selected genes are *ACE2, TMPRSS2, IFITM3, CD209* (DC-SIGN), and *MUC5B*.

The Department of Genetics & Molecular Medicine, Kamineni Hospitals has a genetic counseling and diagnostic facility where patients from different specialties are referred for genetic evaluation. All the patients included in the study were provided with pre- and post-test genetic counseling. Written informed consent to utilize genetic information for research/academic purposes was obtained prior to collecting blood samples.

The variant calling format (vcf) and annotated file data generated from clinical exome studies from individuals/families were stored in a data bank. The selected candidate gene variants were assessed in our internal cohort of 103 individuals, who had earlier provided consent, to perform a pilot study on the susceptibility and disease severity of Indians for COVID-19.

The variant mining flow carried out is briefly described below:

1. Files in vcf format were uploaded on Base Space Variant Interpreter, an Illumina freeware platform. The variants were identified using inbuilt filters.
2. Frequency of the variants was tabulated and compared to global frequencies using TOPMED, EXAC, 1000G databases.

## Results

Data from a total of 103 exomes were analyzed of which 53 were females and 50 were males, and 497 polymorphisms were identified in the five selected candidate genes (Table 1).

**Table 1:** Polymorphisms identified in five genes from preliminary exome data analysis of individuals from India (Note−390 variants in *MUC5B* gene were identified in our cohort, functionally relevant nine are mentioned here, full data can be accessed from Supplementary Data).

**GENE**	***ACE2***	***TMPRSS2***	***CD209***	***IFITM3***	***MUC5B***
TOTAL	17	81	50	49	390- 90 had frequency >10
Upstream	–	9	7	34	3
5′UTR	–	–	–	1	–
EXONIC	1	5	2	2	–
EXON Missense	1	2	2	2	–
INTRONIC	15	59	8	5	4
3′UTR	–	1	20		–
Downstream	–	4	11	5	2

In the *ACE2* gene, 17 polymorphisms were identified of which rs2285666 was the most frequent (9/103), followed by rs113691336, rs971249, and rs4646174, which were observed in 4/103 individuals (Table 2). One missense variant rs4646116 was identified in one individual. This variant causes a protein change lysine to arginine at 26th position, which lies in the extracellular membrane and inhibits interaction with Sar-COV2 Spike protein.

**Table 2:** Showing genewise frequencies of polymorphisms identified with gender stratification (Note−390 variants in *MUC2B* gene were identified in our cohort, functionally relevant nine are mentioned here, full data can be accessed from Supplementary Data).

**rs Number**	**Frequency**	**Male**	**Female**	**Protein change**	**TOPMED**	**1000G**
***ACE2***						
rs4646153	1	1	0	IntronNM_021804.2c.1071-1397G>A	0.6138	
rs113691336	4	2	2	IntronNM_021804.2c.1297+68_1297+69insCTTAT:	0.74378	
rs4646158	4	1	3	IntronNM_021804.2c.1297+68_1297+69insCTTAT		0.8
rs4646165	1	1	0	IntronNM_021804.2c.1443-132G>A-	0.02077	
rs11340646	1	1	0	IntronNM_021804.2c.1443-97delA-		0.0016
rs4646171	1	0	1	IntronNM_021804.2c.1838-552A>G-	0.048906	0.0702
rs4646127	1	1	0	IntronNM_021804.2c.187-2327T>C-	0.716751	0.8093
rs4646174	4	3	1	IntronNM_021804.2c.1896+147G>C-	0.616057	0.4941
rs111691073	1	1	0	IntronNM_021804.2c.1997+520_1997+527delGGAGAGAG-		0.9717
rs233575	2	0	2	IntronNM_021804.2c.2115-625C>T-	0.8633	0.7804
rs971249	4	3	1	IntronNM_021804.2c.584-71A>G-	0.707879	0.6437
rs2048683	1	0	1	IntronNM_021804.2c.584-920A>C-	0.707927	0.6385
rs4646147	1	1	0	IntronNM_021804.2c.901-1231A>T-	0.6146	0.8281
RS4646148	1	0	1	IntronNM_021804.2c.901-380_901-379insTTAA-		0.8283
rs2048684	1	1	0	IntronNM_021804.2c.901-702T>G-	0.744155	0.8283
rs4646116	1	0	1	MissenseNM_021804.2c.77A>Gp.(Lys26Arg)Exon: 2/19	0.0021	0.0046
rs773676270	1	1	0	NM_021804.2c.1164A>Gc.1164A>G(p.(Gln388=))Exon: 10/19	0.0003	0.000016
rs2285666	9	5	4	Splice regionIntronNM_021804.2c.439+4G>A	0.250295	0.3502
***TMPRSS2***						
rs12627374	1	1	0	3-prime UTRNM_001135099.1c.*378G>AExon: 14/14	0.012065	0.059105
rs8128997	1	1	0	Downstream geneNM_001135099.1	0.225838	0.251398
rs460751	1	1	0	Downstream geneNM_001135099.1	0.10353	0.174321
rs397756998	1	0	1	IntronNM_001135099.1c.1186+291dupG	0.10353	0.174321
rs456016	1	0	1	IntronNM_001135099.1c.1187-279A>G	0.054751	0.125799
rs2070787	1	1	0	IntronNM_001135099.1c.1282+446A>C	0.307961	0.293131
rs9974589	1	1	0	IntronNM_001135099.1c.1282+452T>G	0.41055	0.396166
rs2070788	1	0	1	IntronNM_001135099.1c.1282+587C>T	0.41032	0.396765
rs61459778	1	1	0	IntronNM_001135099.1c.1579-343G>C	0.156975	0.136382
rs73905370	1	1	0	IntronNM_001135099.1c.1579-58T>A	0.098146	0.078474
rs386638	1	0	1	IntronNM_001135099.1c.349+1236G>A	0.071865	0.15595
rs2838040	1	1	0	IntronNM_001135099.1c.349+1591T>C	0.328882	0.330871
rs7275220	1	1	0	IntronNM_001135099.1c.349+959C>T		0.47085
rs10154090	1	0	1	IntronNM_001135099.1c.350-2203A>T	0.302657	0.298123
rs555995855	1	1	0	IntronNM_001135099.1c.437-80A>G	0.000096	0.011581
rs573736906	1	1	0	IntronNM_001135099.1c.55+189C>T	0.000096	0.004792
rs7277080	1	1	0	IntronNM_001135099.1c.55+3477G>A	0.331478	0.230232
rs112467088	1	0	1	IntronNM_001135099.1c.55+474T>A	0.277165	0.185703
rs398061769	1	1	0	IntronNM_001135099.1c.556+1040_556+1041delCT		
rs383510	1	1	0	IntronNM_001135099.1c.556+1954A>G		
rs62217531	1	1	0	IntronNM_001135099.1c.556+2420G>A	0.393986	0.297923
rs381179	1	0	1	IntronNM_001135099.1c.556+2679A>G	0.000327	0.000998
rs11701576	1	1	0	IntronNM_001135099.1c.56-146T>C	0.099938	0.16234
rs9974995	1	1	0	IntronNM_001135099.1c.794+188G>A	0.261133	0.260982
rs8131648	1	1	0	IntronNM_001135099.1c.795-587A>G	0.34899	0.444688
rs8131649	1	1	0	IntronNM_001135099.1c.795-590A>G:	0.36706	0.464257
rs8134203	1	1	0	IntronNM_001135099.1c.795-695G>A	0.36706	0.464257
rs8134203	1	0	1	IntronNM_001135099.1c.795-695G>A	0.375701	0.47504
rs2298663	1	0	1	IntronNM_001135099.1c.838+317G>A	0.053182	0.123403
rs2298662	1	0	1	IntronNM_001135099.1c.838+389C>G	0.009939	0.004792
rs61735792	1	1	0	SynonymousNM_001135099.1c.300C>Tc.300C>T(p.(Pro100=)) Exon: 3/14	0.453563	0.422724
rs9981563	1	0	1	Upstream geneNM_001135099.1	0.453595	0.422923/
rs 9981570	1	0	1	Upstream geneNM_001135099.1	0.453579	0.422724
rs9981099	1	0	1	Upstream geneNM_001135099.1		
rs397704299	1	0	1	Upstream geneNM_001135099.1		
rs28707508	1	0	1	Upstream geneNM_001135099.1	0.000111	0.000998
rs460904	2	2	0	Downstream geneNM_001135099.1	0.052824	0.121605
rs455922	2	1	1	IntronNM_001135099.1c.1187-164A>G	0.052601	0.121406
rs462448	2	0	2	IntronNM_001135099.1c.1283-407A>G	0.098465	0.149361
rs743542	2	1	1	IntronNM_001135099.1c.1425+151C>T	0.003106	0.00639
rs457909	2	1	1	IntronNM_001135099.1c.1578+465C>T	0.091584	0.06849
rs3819138	2	1	1	IntronNM_001135099.1c.437-54G>C	0.627572	0.555112
rs56097233	2	1	1	IntronNM_001135099.1c.556+1040_556+1041delCT	0.371862	0.444888
rs365724	2	2	0	IntronNM_001135099.1c.556+1099C>G	0.368486	0.441294
rs365025	2	1	1	IntronNM_001135099.1c.556+1254C>G	0.058311	0.127796
rs2156300	2	1	1	IntronNM_001135099.1c.556+3565C>T	0.380368	0.301917
rs4818239	2	1	1	IntronNM_001135099.1c.794+1024A>G	0.26146	0.261182
rs9636988	2	1	1	IntronNM_001135099.1c.794+1054A>G	0.307093	0.334665
rs9985159	2	1	1	IntronNM_001135099.1c.795-137G>A	0.370381	0.470248
rs2094881	2	1	1	IntronNM_001135099.1c.795-288A>G	0.231691	0.267772
rs2298661	2	1	1	IntronNM_001135099.1c.839-219G>T	0.095287	0.165735
rs465576	2	1	1	NM_001135099.1c.1187-184G>T	0.000008	0.001597
rs542471574	2	1	1	No overlapping canonical transcript	0.348942	0.241014
rs8127674	2	0	2	Upstream geneNM_001135099.1	0.345191	0.238419
rs8129192	2	0	2	Upstream geneNM_001135099.1	0.110578	0.126398
rs4283504	2	1	1	Upstream geneNM_001135099.1	0.306121	0.222644
rs467375	3	2	1	IntronNM_001135099.1c.1186+168C>T		0.82987
rs112132031	3	1	2	IntronNM_001135099.1c.1187-44_1187-43insCCCGAGGCCTTAG	0.213088	0.233427
rs2838042	3	3	0	IntronNM_001135099.1c.349+176A>G	0.265888	0.279553
rs429442	3	3	0	IntronNM_001135099.1c.436+102G>A	0.261356	0.261581
rs9974933	3	3	0	IntronNM_001135099.1c.794+122T>C	0.261436	0.26238
rs9975014	3	3	0	IntronNM_001135099.1c.794+93T>C	0.453571	0.422724
rs8133719	3	2	1	Upstream geneNM_001135099.1	0.308988	0.22524
rs458213	4	3	1	IntronNM_001135099.1c.1011-54A>T	0.302776	0.287141
rs28524972	4	4	0	IntronNM_001135099.1c.1187-101G>C	0.314961	0.426917
rs462326	4	3	1	IntronNM_001135099.1c.1283-130C>G	0.310621	0.307109
rs3787947	4	2	2	IntronNM_001135099.1c.437-153G>A	0.123893	0.162939
rs3787950	4	3	1	SynonymousNM_001135099.1c.336A>Gc.336A>G(p.(Thr112=)) Exon: 3/14	0.052808	0.121406
rs458280	5	3	2	IntronNM_001135099.1c.1011-144A>C	0.36847	0.284545
rs734056	5	3	2	IntronNM_001135099.1c.683+83G>T	0.351634	0.24381
rs75603675	5	3	2	MissenseNM_001135099.1c.23G>Tp.(Gly8Val)Exon: 1/14	0.246982	0.261382
rs12329760	5	2	3	MissenseNM_001135099.1c.589G>Ap.(Val197Met)Exon: 6/14	0.374594	0.444888
rs386416	8	5	3	IntronNM_001135099.1c.437-45C>G	0.461567	0.366214
rs17854725	8	7	1	SynonymousNM_001135099.1c.879T>Cc.879T>C(p.(Ile293=)) Exon: 9/14		
rs17854725	8	5	3	SynonymousNM_001135099.1c.879T>Cc.879T>C(p.(Ile293=)) Exon: 9/14	0.211089	0.209465
rs2298659	8	5	3	SynonymousNM_001135099.1c.888C>Tc.888C>T(p.(Gly296=)) Exon: 9/14		0.82987
rs112132031	9	5	4	IntronNM_001135099.1c.1187-44_1187-43insCCCGAGGCCTTAG	0.054839	0.126198
rs464431	10	7	3	IntronNM_001135099.1c.1011-52T>C	0.375104	0.445288
rs422471	14	8	6	IntronNM_001135099.1c.556+14G>A	0.121966	0.186701
rs140530035	18	9	9	IntronNM_001135099.1c.795-15_795-14delCT		
**CD209**						
rs2287886	28	13	15	Upstream geneNM_021155.3	0.348114	0.412939
rs1544766	26	12	14	3-prime UTRNM_021155.3c.*1202C>TExon: 7/7	0.076899	0.141973
rs4804800	24	13	11	3-prime UTRNM_021155.3c.*2797C>TExon: 7/7	0.20158	0.223243
rs11260028	18	9	9	3-prime UTRNM_021155.3c.*1706T>CExon: 7/7	0.075162	0.139377
rs4804801	17	7	10	3-prime UTRNM_021155.3c.*443A>TExon: 7/7	0.245851	0.328874
rs4804802	15	7	8	3-prime UTRNM_021155.3c.*315T>CExon: 7/7	0.18668	0.216254
rs11260027	14	5	9	Downstream geneNM_021155.3	0.498503	0.468251
rs12460694	13	6	7	3-prime UTRNM_021155.3c.*1670C>TExon: 7/7	0.024656	0.091254
rs11465427	12	5	7	Downstream geneNM_021155.3	0.499753	0.469649
rs7248637	12	8	4	3-prime UTRNM_021155.3c.*898T>CExon: 7/7	0.196611	0.232428
rs7252229	10	5	5	IntronNM_021155.3c.106+11C>G	0.215334	0.198882
rs11465413	10	5	5	3-prime UTRNM_021155.3c.*1974T>AExon: 7/7	0.188265	0.219249
rs11465406	10	3	7	3-prime UTRNM_021155.3c.*1695delGExon: 7/7	0.809036	0.747404
rs11465391	9	4	5	IntronNM_021155.3c.1014-37C>G	0.161052	0.147165
rs6603119	8	3	5	3-prime UTRNM_021155.3c.*722A>GExon: 7/7	0.352908	0.425319
rs11465421	8	4	4	3-prime UTRNM_021155.3c.*2629C>AExon: 7/7	0.486278	0.47484
rs11465410	7	4	3	3-prime UTRNM_021155.3c.*1846A>GExon: 7/7	0.07615	0.102236
rs11465409	7	4	3	3-prime UTRNM_021155.3c.*1839C>TExon: 7/7	0.077575	0.104034
rs11465408	7	4	3	3-prime UTRNM_021155.3c.*1771C>TExon: 7/7	0.075107	0.101837
rs7248772	6	4	2	3-prime UTRNM_021155.3c.*754T>CExon: 7/7	0.33753	0.394369
rs11465411	6	4	2	3-prime UTRNM_021155.3c.*1848T>CExon: 7/7	0.076269	0.102236
rs76638576	4	4	0	Upstream geneNM_021155.3	0.069644	0.055312
rs8105572	4	0	4	IntronNM_021155.3c.901-222G>A	0.15367	0.141374
rs8105483	4	0	4	IntronNM_021155.3c.901-178G>C	0.153733	0.142173
rs11465384	3	0	3	IntronNM_021155.3c.749-28C>T	0.059402	0.031949
rs11465371	3	1	2	IntronNM_021155.3c.106+246G>T		0.03574
rs34472423	3	1	3	Downstream geneNM_021155.3	0.694667	0.665335
rs7254342	3	1	2	Downstream geneNM_021155.3	0.241104	0.253395
rs1291625799	3	1	2	missense p.Gln191Leu		
rs4804803	2	0	2	Upstream geneNM_021155.3	0.255511	0.233427
rs78866372	2	2	0	SynonymousNM_021155.3c.993C>Tc.993C>T(p.(Asp331=)) Exon: 6/7	0.007175	0.004393
rs553008652	2	0	2	SynonymousNM_021155.3c.1098C>Tc.1098C>T(p.(Asp366=)) Exon: 7/7	0.000048	0.000399
rs17438280	2	0	2	Intron	0.070607	0.064497
rs4045399	2	1	1	Downstream geneNM_021155.3	0.694667	0.665335
rs796471429	2	1	1	Downstream geneNM_021155.3		
rs7248227	2	2	0	Downstream geneNM_021155.3	0.464402	0.435903
rs1981837	2	1	1	Downstream geneNM_021155.3	0.305181	0.334665
rs11465397	2	0	2	3-prime UTRNM_021155.3c.*675C>TExon: 7/7	0.129523	0.100839
rs544874106	2	1	1	3-prime UTRNM_021155.3c.*625C>TExon: 7/7	0.000056	0.000399
rs56062941	1	0	1	Upstream geneNM_198492.3	0.28962	0.336462
rs796136624	1	0	1	Upstream geneNM_198492.3		
rs12610438	1	0	1	Upstream geneNM_198492.3	0.259963	0.311901
rs11881682	1	0	1	Upstream geneNM_021155.3	0.172393	0.147764
rs565809638	1	1	0	IntronNM_021155.3c.900+91G>T	0.000056	0.004792
rs56341602	1	0	1	Downstream geneNM_021155.3		0.6847
rs7258175	1	0	1	Downstream geneNM_021155.3	0.464537	0.435903
rs183145639	1	1	0	Downstream geneNM_021155.3	0.003241	0.001997
rs11465396	1	0	1	3-prime UTRNM_021155.3c.*467T>CExon: 7/7	0.045067	0.031949
rs879074669	1	1	0	3-prime UTRNM_021155.3c.*2523G>AExon: 7/7		
rs141131967	1	1	0	missense p.Arg154Gly		0.004393
**IFITM3**						
rs3888188	5	3	2	Upstream gene NM_021034.2		
rs61876247	5	5	0	Upstream gene NM_021034.2		
rs61876248	6	6	0	Upstream gene NM_021034.2		
rs28447048	7	6	1	Upstream gene NM_021034.2		
rs56232455	3	1	2	Upstream gene NM_021034.2		0.17073
rs7479267	20	11	9	Upstream gene NM_021034.2		0.47125
*rs71452596*	19	9	10	Upstream gene NM_021034.2		*0.479*
*rs7478728*	18	9	9	Upstream gene NM_021034.2		*0.47903*
rs10902122	12	7	5	Upstream gene NM_021034.2		0.0573
rs55809726	9	6	3	Upstream gene NM_021034.2		0.32248
rs77263314	7	5	2	Upstream gene NM_021034.2		0.32248
rs11821786	7	5	2	Upstream gene NM_021034.2		0.32248
rs11246066	2	2	0	Upstream gene NM_021034.2	0.209639	0.252995
rs7931303	4	4	0	Upstream gene NM_021034.2	0.494537	0.423722
rs6598043	1	1	0	Upstream gene NM_021034.2	0.492379	0.42512
rs6598042	1	1	0	Upstream gene NM_021034.2	0.295561	0.170727
rs6598045	10	5	5	Upstream gene NM_021034.2		0.21346
rs11828350	5	4	1	Upstream gene NM_021034.2		0.3115
rs10794307	1	0	1	Upstream gene NM_021034.2	0.487099	0.441693
rs112701542	1	0	1	Upstream gene NM_021034.2	0.07971	0.053514
rs9666598	4	4	0	Upstream gene NM_021034.2	0.144878	0.116214
rs7948108	1	0	1	Upstream gene NM_021034.2	0.441936	0.398762
rs60016595	2	2	0	Upstream gene NM_021034.2		0.42851
rs3215389	5	2	3	Upstream gene NM_021034.2		
rs56228238	3	3	0	Upstream gene NM_021034.2		
rs55671406	2	1	1	Upstream gene NM_021034.2		
rs35218683	4	2	2	Upstream gene NM_021034.2		
*rs28602580*	2	1	1	Upstream gene NM_021034.2		
rs35409983	3	2	1	Upstream gene NM_021034.2		
rs9666637	1	0	1	Upstream gene NM_021034.2	0.202918	0.29393
rs7478728	1	0	1	Upstream gene NM_021034.2		0.47903
rs9666295	1	1	0	Upstream gene NM_021034.2	0.093933	0.0623
rs77776420	1	1	0	Upstream gene NM_021034.2		
rs116991140	1	1	0	Upstream gene NM_021034.2	0.028224	0.019169
rs12252	12	10	2	Synonymous NM_021034.2 c.42T>C c.42T>C(p.(Ser14=)) Exon: 1/2	0.155223	0.236422
rs11553885	8	6	2	Synonymous NM_021034.2 c.165C>T c.165C>T(p.(Pro55=)) Exon: 1/2	0.000064	
rs1136853	7	5	2	Missense NM_021034.2 c.9C>A p.(His3Gln) Exon: 1/2	0.041882	0.039337
rs199749095	9	5	4	Missense NM_021034.2 c.208C>A p.(Pro70Thr) Exon: 1/2	0.000008	
rs28688930	34	21	16	Intron NM_021034.2 c.250-247G>C	0.101793	
rs28655829	37	23	14	Intron NM_021034.2 c.250-176A>G	0.082593	0.058107
rs3835195	37	22	15	Intron NM_021034.2 c.250-125C>T	0.109558	0.083067
rs746767660	3	3	0	Intron NM_021034.2 c.249+174_249+175delTG	0.000295	
rs6421983	14	7	7	Intron NM_021034.2 c.249+171G>A	0.309673	0.164936
rs77612739	1	0	1	Downstream gene NM_021034.2, Downstream gene NM_003641.3	0.087817	0.071286
rs9666182	1	1	0	Downstream gene NM_021034.2, Downstream gene NM_003641.3	0.207712	0.134784
rs7938456	1	1	0	Downstream gene NM_021034.2, Downstream gene NM_003641.3	0.489862	0.488818
rs12421894	1	1	0	Downstream gene NM_021034.2, Downstream gene NM_003641.3	0.089513	0.072284
rs7947900	1	1	0	Downstream gene NM_021034.2, Downstream gene NM_003641.3	0.306153	0.160743
rs34481144	23	12	11	5-prime UTR NM_021034.2 c.-23G>A Exon: 1/2	0.309768	0.178115
**MUC5B**						
rs2672794	1	1	0	Upstream geneNM_002458.2	0.311035	0.313299
rs56235854	2	1	1	Upstream geneNM_002458.2	0.042662	0.03734
rs7115457	3	1	2	Upstream geneNM_002458.2	0.130543	0.190895
rs2735727	12	5	7	IntronNM_002458.2c.2881-157G>A	0.423364	0.457867
rs12417955	4	2	2	Downstream geneNM_002458.2	0.459767	0.428514
rs56367042	3	2	1	Downstream geneNM_002458.2	0.051876	0.052915
rs2735733	31	17	14	IntronNM_002458.2c.3970+35C>T	0.428007	0.461861
rs2249073	26	14	12	IntronNM_002458.2c.15045+79T>C	0.478139	0.454073
rs2857476	28	18	10	IntronNM_002458.2c.16801-59T>C	0.455227	0.422724

In *TMPRSS2* gene 81 variants were identified and the most common polymorphism identified was rs140530035 (18/103), followed by rs422471 (14/103) and rs464431 (10/103). Both missense variants were in the non-catalytic domain, which does not interact directly with SAR-COV2 S protein (Table 2).

In the *CD209* gene, 50 variants were identified, and the most frequent ones were rs2287886 (28/103), rs1544766 (26/103), and rs4804800 (24/103) followed by rs11260028 (18/103), rs4804801 (17/103), rs4804802 (15/103), rs11260027 (14/103), and rs12460694 (13/103) (Table 2). Both missense variants rs1291625799 (3/103) causing p.Gln191Leu and rs141131967 (1/103) causing p.Arg154Gly alter disulfide formation in the C-type lectin domain, which functions as a calcium-dependent carbohydrate-recognition domain, were identified in our cohort.

A total of 49 variants were identified in the *IFITM3* gene. Most frequent variants identified were rs28655829 (37/103), rs3835195(37/103), rs28688930 (34/103), rs34481144 (23/103), rs7479267 (20/103), rs71452596 (19/103), rs7478728 (18/103), rs6421983 (14/103), rs10902122 (12/103), rs12252 (12/103), and rs12252 (10/103).

*MUC5B* gene exhibited the maximum number of variants. Of the total 390 variants identified, 93 of them had frequency of above 10, the most frequent variants observed were rs2735733 (31/103), rs2249073 (26/103), and rs2857476 (28/103). Polymorphisms rs2735727 (12/103) and rs12417955 (4/103) that lead to alternative splicing of *MUC5B* and rs56367042 (3/103) are speculated to be involved in the pathogenesis of idiopathic pulmonary fibrosis.

## Discussion

A wide range of inter individual variation is being observed in the infectivity, disease symptoms, progression of disease, and mortality of COVID-19 between different populations. Host genetic factors have frequently been implicated in respiratory infectious diseases, single nucleotide polymorphisms (SNPs), or commonly known gene polymorphisms have been considered responsible for both ethnic and inter individual variation (Patarčić et al., 2015). A recent twin study indicated that there is 50% genetic heritability in response to the SARS-CoV2 infection justifying the need to evaluate sequence variants in candidate genes (Williams et al., 2020). Until exome and genome data of individuals with specific SARS-CoV2 infection, unaffected contacts and individuals with range of disease symptoms, as well as, those who succumbed to disease is available, analysis of existing data from random population is the only option to identify variants, which may help to develop a polymorphism panel to identify individuals who are susceptible to infection and also those at risk of developing severe COVID-19 disease.

Since host genetic polymorphisms have been demonstrated to be associated with vulnerability to human infection, in this study five candidate genes—*ACE2, TMPRSS2, CD209, IFITM3*, and *MUC5B*—were selected based on their relevance to the current pandemic. All variants reported from each gene were identified from the sequence data available with us as vcf files. The data belonged to 103 individuals of Indian origin (who had consented for use of their data for research) and was selected randomly without any prior bias. Maximum number of variants (*n* = 390) were identified in *MUC5B* gene and the least (*n* = 17) in *ACE2* gene.

*ACE2* is a human homolog for ACE, which is well-known for its role in the Renin-Angiotensin pathway. *ACE2* was identified in the year 2000, it consists of 805 amino acids, and it is a type I transmembrane glycoprotein with a single extracellular catalytic motif (Kuba et al., 2010). It is a carboxypeptidase that catalyzes liberation of vasodilator peptide, angiotensin, from angiotensin II, thus is responsible for counterbalancing the potent vasoconstrictor effects of angiotensin II (Schindler et al., 2007). It also has various other physiological functions, like being a key regulator of dietary amino acid homeostasis in colitis (Hashimoto et al., 2012). But in the context of COVID-19, *ACE2* is the major viral receptor and is important for SARS-CoV-2 entry into the cell, making it extremely relevant for infectivity.

Several polymorphisms in *ACE2* were reported from a large commercial dataset (Cai, 2020). A recent study from Italian population states that *ACE2* variants underlie interindividual variability and susceptibility to COVID-19 (Hashimoto et al., 2012). In our analysis, 17 variants were identified, and the earlier reported missense variant rs4646116, which is responsible for Lys26Arg change in exon 2, was seen with a frequency of 0.0048 in our preliminary data. The frequency of rs 4646116 is reported as 0.0021 in TOPMED and 0.0046 in 1000 Genomes, it has been reported from most populations with Europeans having the highest frequency (Asselta et al., 2020). Another intronic polymorphism rs4646171 was identified with a frequency of 0.0048 and was reported with a higher frequency of 0.048 in TOPMED and 0.070 in 1000 Genome data. The most common polymorphism in our cohort was rs2285666, which exhibited a frequency of 0.0436, which was higher than what was observed in the two databases (Table 2). The A/A genotype of rs2285666 has a 50% lower expression level of *ACE2* compared to G/G genotype and may be protective (Asselta et al., 2020).

Earlier studies reported that the A allele of *ACE2* rs4646127 intronic SNP is responsible for decreased tissue expression of *ACE2* and was considerably more common in people of European (44.1%) descent and less common in people of East Asian (1%) descent. Another polymorphism rs4646174 has been associated with central pulse pressure, Brain Natriuretic Peptide, and NYHA classification of patients with chronic heart failure (Malard et al., 2013). In several studies, different *ACE2* polymorphisms with altered *ACE2* expression have been associated with systolic blood pressure, diabetes, cardiovascular disorders, stroke, etc. These could be the likely reason behind the individuals with comorbidities succumbing severely to the COVID-19 infection than others.

*TMPRSS2* is considered to play a role in SARS-CoV-2 virus entry in human cells along with *ACE2*. The host cell protease *TMPRSS2* is involved in the fusion of the virus with cell membrane, thereby it is important to understand the role of its variants in COVID-19 infection and disease progression (Bertram et al., 2013; Heurich et al., 2014). *TMPRSS2* is known to cleave the influenza A virus and knock out *TMPRSS2*^−/−^ mice are resistant to infection, indicating the importance of this gene in the spread and pathogenesis of viral infection (Lambertz et al., 2020).

The role of *TMPRSS2* gene in prostate cancer is well-known; however, there are very few reports about the association of *TMPRSS2* polymorphism with respiratory distress. An SNP of the *TMPRSS2* gene (rs12329760 C>T; Met160Val) present in an exonic splicing enhancer srp40 site, which is highly conserved across mammals, has been found to be associated positively with *TMPRSS2*–*ERG* fusion by translocation due to an increased chance of exon skipping in prostate cancer (Bhanushali et al., 2018). This rs12329760 polymorphism was seen in 4.85% of individuals from our study.

However, *TMPRSS2* gene eQTLs in lungs appear to cluster at the 3′ end and are potentially associated with expression of alternative transcripts in lungs. Amongst these common eQTLs, HaploReg annotations indicated rs 4818239 as an important SNP (Sharma et al., 2020). This was identified in about 2% of the individuals in our population. A recent Indian paper assessed the *in-silico* functional analysis of various variants some of which were identified in our cohort, like rs4818239 (2/103), rs734056 (5/103), and rs62217531 (1/103) (Sharma et al., 2020). It has been demonstrated that genetic variants with higher *TMPRSS2* expression confer greater risk to severe influenza A(H1N1). Notably, rs2070788 and rs383510 had high expression of *TMPRSS2* and were significantly associated with the susceptibility to Influenza A(H7N9) (Cheng et al., 2015). The rs2298662 showed high LD with rs2070788 associated with respiratory disorders (Cheng et al., 2015). All these three variants—rs2070788, rs2298662 (1/103), and rs383510 (1/103)—were found in our study at a low frequency. The same variants also increase susceptibility to human Influenza A(H7N9) and may be relevant for COVID-19 infectivity. A haplotype, predicted to be associated with higher *TMPRSS2* expression, is characterized by three SNPs (rs2070788, rs9974589, rs7364083), whose Minor Allele Frequency (MAF) is significantly increased in Europeans and was 9% higher in Italians with respect to East Asians (Asselta et al., 2020). The polymorphism rs9974995 is nominally associated with phenotype related to respiratory function or respiratory medication (Salmeterol or fluticasone propionate) (Wang et al., 2020).

Higher expression of *ACE2* and *TMPRSS2* in males, African Americans, and patients with diabetes mellitus provides rationale for monitoring these subgroups for high infectivity and poor COVID-19 outcomes. The lower expression of *ACE2* and *TMPRSS2* with inhaled corticosteroid use warrants prospective study of inhaled corticosteroid use as a predictor of decreased susceptibility to SARS-CoV-2 infection and decreased COVID-19 morbidity (Peters et al., 2020). The two polymorphisms rs112132031 and rs75603675 identified in 10.67 and 4.85% of our cohort, respectively, may be relevant for COVID-19 Cytokine Release syndrome and conjunctival infection according to earlier reports (Peters et al., 2020).

The *CD209* gene, which encodes the DC-SIGN (dendritic cell-specific intercellular adhesion molecule-3-grabbing non-integrin), a key effector of the innate immunity and antiviral defense, is a receptor expressed in the dendritic cells involved in recognition of oligosaccharides present in several pathogens (Granelli-Piperno et al., 2005). Hence, polymorphisms in this gene may explain susceptibility/resistance to infection as well as severity of symptoms in several infectious diseases. In our cohort, 50 variants have been identified in this gene. The GG genotype of rs2287886 present in the promoter region of *CD209* was identified in 27.18% individuals in our cohort. It is reported to be associated with development of dengue fever requiring hospitalization, cytomegalovirus disease after allogeneic stem-cell transplantation, predisposition to developing tick-borne encephalitis, invasive pulmonary Aspergillosis infection, Kawazaki infection, and colorectal cancer (Mezger et al., 2008; Barkhash et al., 2012; Sainz et al., 2012; Alagarasu et al., 2013; Lu et al., 2013; Portman et al., 2013; Czupryna et al., 2017). Individuals with genotype AA at rs2287886, whose frequency in Central Asian Mongoloids is high, have shown to express higher levels of *CD209*, thereby having a higher rate of infection by cytomegalovirus than DCs carrying the GG genotype creating an analogous situation for the existence of Epstein-Barr virus (EBV), since glycoproteins on the viral surface, which are conserved are similar with those on cytomegalovirus (Barkhash et al., 2012). *CD209* is also known to transport viruses via immature DCs from the periphery to lymph nodes, where CD4 cells get activated and infected, elucidating a link between *CD209* genetic variation and CD4 count. In our dataset, the rs8105483 was observed in 3.88% subjects and rs2287886 in 27.18%, which together form a protective haplotype, which has a higher CD4 count (Geijtenbeek et al., 2003; Tailleux et al., 2003; Tassaneetrithep et al., 2003; Barreiro et al., 2006; Hennig et al., 2011). These polymorphisms may similarly be protective for COVID-19.

In addition, significant associations were found between high risk of Kawasaki disease with *CD209* polymorphisms rs4804800 and rs2287886 (Kuo et al., 2014). A study by Ovsyannikova et al. (2011) has shown associations between promoter SNP rs11881682 and intronic SNPs: rs8105572 and rs7252229 of the *CD209* gene and measles-specific IFN-γ Elispot responses in Caucasian subjects (Ovsyannikova et al., 2011). SNPs in *CD209* rs4804800, rs11465384, rs7248637, and rs7252229 have shown association with an increased risk to develop invasive pulmonary aspergillosis infection (Sainz et al., 2012). The polymorphism rs7248637 in *CD209*, which showed association with dengue in the Colombian population (Avendaño-Tamayo et al., 2019), was observed in 11.65% subjects in our dataset, and 9.7% subjects in our dataset showed rs11465413 associated with atopic sensitization (Penders et al., 2010).

Interferon-induced membrane protein that inhibits the entry of viruses into the host cell cytoplasm by preventing viral fusion with cholesterol depleted endosomes is encoded by the *IFITM3* gene (Zhao et al., 2019). It has a capacity to inactivate new enveloped viruses, which bud out of the infected cell. It has been shown to be active against multiple viruses, including influenza A virus, SARS coronavirus (SARS-CoV), Ebola virus (EBOV), Dengue virus (DNV), human immunodeficiency virus type 1 (HIV-1), etc. (Brass et al., 2009; Lu et al., 2011). Pathways through which *IFITM3* functions are: Innate Immune System and Interferon gamma signaling. Studies have shown that the first 21 amino acids of the N-terminus of *IFITM3* gene are required for attenuation of vesicular stomatitis virus replication, and that truncated *IFITM3* protein fails to restrict the replication of various strains of influenza virus, as well as HIV-1 (Jia et al., 2012; Bailey et al., 2014). Williams et al. (2014) and Kim et al. (2019) showed that even full-length *IFITM3* restricts entry and replication of H1N1. Polymorphisms of this gene have been studied in several infections. The polymorphism rs6598045 c.−188T > C (4.85% in our cohort) induces a difference in the binding capacity of the transcription factor causing a difference in the transcription efficiency of the *IFITM3* gene, which was reported to exhibit a strong association with influenza H1N1 2009 pandemic virus infection (Shen et al., 2013). Another functional polymorphism rs3888188, showed that peripheral-blood mononuclear cells carrying GG genotype had reduced *IFITM3* mRNA level compared to those with TT or GT genotype, which predisposes toward pulmonary tuberculosis in Iranian and Han Chinese populations (Shen et al., 2013).

Previous studies predicted that rs12252 C allele might produce an alternate spliced transcript that encodes an aberrant truncated protein Δ21 of *IFITM3*, which reduces the cellular resistance to influenza viruses by blocking early stage of viral replication (Everitt et al., 2012; Compton et al., 2016). Association of this polymorphism has been observed in Chinese population with pandemic influenza (H1N1 09pdm), seasonal influenza (H3N2 and influenza B), and avian influenza (H7N9) (Carter et al., 2018). In our cohort, 11.65% of the individuals carried this allele. Seasonal influenza hospital admissions were associated with rs7948108, which was observed with a low (0.97%) percentage in our cohort.

The rs34481144 is considered to directly affect *IFITM3* promoter function. The risk allele A is linked to diminished promoter activity by increasing the binding of the methylation-sensitive CTCF transcriptional repressor. This ablation of methylation site controls *IFITM3*-promoter methylation in memory Cytotoxic T Lymphocytes (CTLs) reducing CTCF binding to increase *IFITM3* expression, which leads to increased memory CTL survival and more efficient viral clearance from infected airways (Eisfeld and Kawaoka, 2017). This was observed in 22% of individuals of our cohort and may be a relevant variant for SARS-CoV-2 clearance and reduced progression of disease.

*MUC5B* gene encodes a member of the mucin family of proteins, which are highly glycosylated macromolecular components of mucus secretions. This family member is the major gel-forming mucin in mucus (Ridley and Thornton, 2018). It is a major contributor to the lubricating and viscoelastic properties of whole saliva, normal lung mucus, and cervical mucus. This gene has been found to be upregulated in some human diseases, while pathogenic variants have been reported to cause pulmonary fibrosis—a lung disease characterized by shortness of breath and varying degrees of inflammation and fibrosis, which is rapidly progressive and acute lung injury with subsequent scarring and end-stage lung disease (Zhang et al., 2019). Many of these symptoms are similar to that reported in COVID-19 disease.

Airway lining mucus serves as the first line of defense during upper respiratory infection. Pathogens trapped in the mucus layer are first removed by the mucociliary clearance mechanism of the underlying airway epithelium as well as macrophages and then by neutrophils recruited into the airways in response to inflammatory mediators released by epithelial cells and macrophages (Kim, 2012). Adult *MUC5B*-deficient mice displayed bronchial hyperplasia and metaplasia, interstitial thickening, alveolar collapse, immune cell infiltrates, fragmented and disorganized elastin fibers, and collagen deposits that were, for approximately one-fifth of the mice, associated with altered pulmonary function leading to respiratory failure demonstrating that the mouse *MUC5B* is essential for maintaining normal lung function (Valque et al., 2019).

*MUC5B* gene had the maximum number (*n* = 390) of variants in our cohort. The SNP rs2672794 is associated significantly with increased susceptibility to coal workers' pneumoconiosis in a Chinese population (Ji et al., 2014). While the rs56235854 polymorphism is associated with severe asthma (Johnson, 2020), this was identified in 1.94% of individuals analyzed in our dataset. The *MUC5B* gene rs2735733, rs2249073, and rs2857476 were associated with dental caries; all the three variants were present in 30.09, 25.24, and 27.18%, respectively, of individuals from our cohort, indicating that Indians are highly susceptible to dental caries.

Polymorphisms rs2735727 (12/103) and rs12417955 (4/103) that lead to alternative splicing of *MUC5B* and rs56367042 (3/103) are speculated to be involved in the pathogenesis of idiopathic pulmonary fibrosis (Nance et al., 2014). A promoter variant rs7115457 is associated with diffuse pan bronchiolitis (Kamio et al., 2005), while another *MUC5B* promoter polymorphism, rs35705950, is the strongest risk factor, genetic or otherwise, accounting for 30–35% risk of developing Idiopathic pulmonary fibrosis (IPF), a disease that was previously considered idiopathic. This *MUC5B* variant can potentially be used to identify individuals with preclinical pulmonary fibrosis and is predictive of radiologic progression of disease. The excessive production of *MUC5B* either enhances injury due to reduced mucociliary clearance or impedes repair consequent to disruption of normal regenerative mechanisms in the distal lung (Evans et al., 2016). This variant rs35705950 (1000G frequency T = 0.0467), was not identified in our cohort and maybe responsible for protecting us from COVID-19 full-blown symptoms.

A total of 497 polymorphisms were identified in five genes in 103 exomes analyzed; 38 polymorphisms identified in our cohort have been reported earlier in literature and have functional significance. Two polymorphic variants rs35705950 of *MUC5B* that increase susceptibility to pulmonary fibrosis and the common “European” haplotype of *TMPRSS2* gene (composed of SNPs rs463727, rs34624090, rs55964536, rs734056, rs4290734, rs34783969, rs11702475, rs35899679, and rs35041537) are totally absent in the Asian population. This may be one of the plausible factors for reduced severity of COVID-19 in Asians compared to Europeans.

Based on the function and expression, we categorized the 38 polymorphisms into three groups: polymorphisms increasing susceptibility to the infection (respiratory illnesses), polymorphisms that confer enhanced immunity, and the polymorphisms that are involved in other pathologies, which can induce comorbidities and make an individual fall under high risk category. The six protective polymorphisms, three in *ACE2*, two in *CD209*, and one in *IFITM3* genes (Table 3) were identified in our cohort, at least one was present in 38.83% of the individuals analyzed. There were 27 risk susceptibility polymorphisms identified in four genes (Table 3) and cumulative count of individuals with at least one risk polymorphism was 47.57%. There were five polymorphisms, three in *MUC5B* and one each in *ACE2* and *TMPRSS2* genes, which are associated with comorbidities and the cumulative count of individuals with at least one risk polymorphism in our cohort was 40.77% (Table 3).

**Table 3:** Showing stratification of polymorphisms into protective, risk inducing, and associated with comorbidities.

	***ACE2***	***TMPRSS2***	***CD209***	***IFITM3***	***MUC5B***
Protective	rs4646116 rs464612 rs2285666		rs2287886 rs8105483	rs34481144	
Risk/Susceptibility		rs12329760 rs4818239, rs62217531, rs75603675, rs2298662, rs2070788 rs383510, rs9974589, rs997499	rs11881682, rs8105572, rs7252229, rs11465384, rs7248637, rs1146541	rs7948108, rs12252, rs4804800 rs4804803, rs6598045, rs3888188	rs2672794, rs56235854, rs7115457, rs2735727, rs12417955, rs56367042
Comorbidities	rs4646174	rs112132031			rs2735733, rs2249073 rs2857476

A larger study for validating our results from Indian population is required, and the sequence data for this maybe already available with the Council of Scientific and Industrial Research—Institute of Genomics and Integrative Biology (CSIR-IGIB) consortium and commercial companies doing testing for Indian patients since 2014. These will also include individuals from different regions of India, unlike the present study where the majority of individuals were from South India. Preliminary sequence data analysis results from five selected candidate genes presented in this paper highlights the importance of identifying polymorphisms from COVID-19 infected asymptomatic and symptomatic patients to give more meaningful results, which will help in managing this pandemic.

## Conclusion

This is the first study from an Indian population presenting polymorphisms from five selected candidate genes, which may be important for understanding the infectivity and progression of COVID-19 in our population. A larger dataset needs to be analyzed to validate the results and develop a panel of polymorphisms useful for identifying individuals at risk, as well as, those likely to have severe disease symptoms.

## Data Availability Statement

The original contributions presented in the study are included in the article/Supplementary Material, further inquiries can be directed to the corresponding author/s.

## Ethics Statement

Ethical review and approval was not required for the study on human participants in accordance with the local legislation and institutional requirements. Written informed consent to participate in this study was provided by the participants' legal guardian/next of kin.

## Author Contributions

GI co-conceptualized the study, performed data analysis, and wrote the first draft of the manuscript. SS compiled and analyzed the data. SZ co-conceptualized the study and assisted in scientific editing of the manuscript. DS, VM, SP, AS, NA, and NB co-compiled the data. AN co-conceptualized the study. QH conceptualized, supervised the study, and performed scientific editing of the manuscript. All authors approved the final draft of the manuscript and agree to be accountable for the content of the work.

## Conflict of Interest

The authors declare that the research was conducted in the absence of any commercial or financial relationships that could be construed as a potential conflict of interest.
